# Family Cataract

**Published:** 1840-09

**Authors:** 


### FAMILY CATARACT.

Although cataract is not unknown as a family disease, we have thought the following example worthy of being recorded.

Near Chillicothe, in Ohio, there lives a family by the name of Bunn, in which five cases of that disease have occurred among nine children. We shall say a few words of each, beginning with the oldest.

Hannah, the oldest, near 36, with hazle eyes and reddish chestnut hair, when 25, presented in the centre of her left eye a small white speck, which gradually enlarged till she could only see objects obliquely. About three years after it commenced the sight of the other began to fail. Twelve months ago we found them both cataracts, with capsular opacity. The left eye was weak and we operated on it without subsequent inflammation. A second operation has lately been found necessary, and is likely to be successful.

2. Mary, aged thirty-four, has dark hazle eyes, and hair nearly black—sight good.

3. David, next in time, now twenty-eight, with hazle eyes, began to lose his sight at seventeen, two years afterwards applied to us in Cincinnati, with capsular cataract. A single laceration, not followed by inflammation, removed it.

4. Sarah Ann, now twenty-four, with hazle eyes, and chesnut colored hair, when nine years old, was discovered to have "something" in the pupil of the left eye. In two years she was nearly blind in both eyes. When she was fourteen years old, the capsules of both eyes were successfully lacerated by Dr. French. When the eyes of this patient, the pupil being somewhat dilated, are looked into obliquely it may be seen that the posterior capsule was opaque; the outer ends of the white radiating bands still remaining there.

Nancy with black hair and eyes, died at the age of twenty-two, with good eye sight.

5. Ezekiel, now twenty-two, with hazle eyes and dark hair, when eighteen, observed some failure of sight; and discovered, while he could still see to shoot with a rifle, that he missed the object—the ball invariably passing to the left of the object. When it was fifteen yards off, the ball would generally strike about five inches from it. From the beginning, his eyes although uninflamed were somewhat intolerant of light. In the summer of 1839, we found each eye with central opacity and opake bands radiating from it, the latter in the posterior capsule. Sight much reduced. The capsule of the right eye was lacerated without subsequent inflammation. Two months afterwards, from cold, a severe and protracted inflammation occurred, and we lately found a large fragment of the capsule, *attached* to the outer pupillary margin of the iris, and projecting into the axis of vision. It has been removed, and his sight restored—no inflammation having followed.

6. Joseph aged nineteen, has chesnut hair and hazle eyes—sight unimpaired.

7. Elizabeth aged seventeen years, with hazle eyes and dark brown hair, has intolerance of light, dimness of vision, and the appearance of cataract forming in both eyes.

8. Abraham, aged thirteen, has hair of a flaxen color, and eyes flaxen grey. His sight said not to be very good.

9. Clarissa, the youngest, nine years old, has eyes nearly black, and hair becoming so. Sight good.

The father of these children, now dead, had good vision. Eyes flaxen grey, hair black.

The mother, of a sanguine temperament, has both eyes and hair of a very dark hazle. Her sight is good. Her mother had blue eyes and red hair—her father black hair and eyes. From this union seems to have resulted the hazle chesnut and auburn hue of the coloring matter of the iris and hair of the mother and children. The mother's sight, however, is good; and no case of cataract is known to have existed among her ancestors, or her husband's. All the children are well developed and healthy. D.

